# Supplementary material for: A fault-intrusion-tolerant system and deadline-aware algorithm for scheduling scientific workflow in the cloud
Source: PeerJ Comput Sci. 2021 Nov 2;7:e747. doi: 10.7717/peerj-cs.747 (PMC8576554; doi:10.7717/peerj-cs.747)
Supplement: Supplemental Information 6 [file peerj-cs-07-747-s006.zip › Myproject1.1/docs/org/workflowsim/class-use/Task.html]

Uses of Class org.workflowsim.Task


JavaScript is disabled on your browser.


- Overview
- Package
- Class
- Use
- Tree
- Deprecated
- Index
- Help

- Prev
- Next

- Frames
- No Frames

- All Classes

## Uses of Class org.workflowsim.Task

- Packages that use Task

  | Package | Description |
  | --- | --- |
  |  |  |
  | --- | --- |
  | org.workflowsim |  |
  | org.workflowsim.clustering |  |
- - ### Uses of Task in org.workflowsim

    Subclasses of Task in org.workflowsim

    | Modifier and Type | Class and Description |
    | --- | --- |
    |  |  |
    | --- | --- |
    | `class` | `Job` Job is an extention to Task. |

    Fields in org.workflowsim with type parameters of type Task

    | Modifier and Type | Field and Description |
    | --- | --- |
    |  |  |
    | --- | --- |
    | `protected java.util.Map<java.lang.String,Task>` | WorkflowParser.`mName2Task` Map from task name to task. |
    | `protected java.util.List<Task>` | ClusteringEngine.`taskList` The task list |
    | `protected java.util.List<Task>` | WorkflowPlanner.`taskList` The task list. |
    | `protected java.util.List<? extends Task>` | ClusteringEngine.`taskReceivedList` The task received list. |
    | `protected java.util.List<? extends Task>` | ClusteringEngine.`taskSubmittedList` The task submitted list. |

    Methods in org.workflowsim that return types with arguments of type Task

    | Modifier and Type | Method and Description |
    | --- | --- |
    |  |  |
    | --- | --- |
    | `java.util.List<Task>` | Task.`getChildList()` Gets the child list of the task |
    | `java.util.List<Task>` | Task.`getParentList()` Gets the list of the parent tasks |
    | `java.util.List<Task>` | ClusteringEngine.`getTaskList()` Gets the task list. |
    | `java.util.List<Task>` | Job.`getTaskList()` Gets the list of tasks in this job |
    | `java.util.List<Task>` | WorkflowParser.`getTaskList()` Gets the task list |
    | `java.util.List<Task>` | WorkflowPlanner.`getTaskList()` Gets the task list. |
    | `java.util.List<Task>` | ClusteringEngine.`getTaskReceivedList()` Gets the task received list. |
    | `java.util.List<Task>` | ClusteringEngine.`getTaskSubmittedList()` Gets the tasks submitted list. |

    Methods in org.workflowsim with parameters of type Task

    | Modifier and Type | Method and Description |
    | --- | --- |
    |  |  |
    | --- | --- |
    | `void` | Task.`addChild(Task task)` Adds a task to existing child list |
    | `void` | Task.`addParent(Task task)` Adds a task to existing parent list |

    Method parameters in org.workflowsim with type arguments of type Task

    | Modifier and Type | Method and Description |
    | --- | --- |
    |  |  |
    | --- | --- |
    | `protected void` | ClusteringEngine.`setTaskList(java.util.List<Task> taskList)` Sets the task list. |
    | `protected void` | WorkflowParser.`setTaskList(java.util.List<Task> taskList)` Sets the task list |
    | `protected void` | WorkflowPlanner.`setTaskList(java.util.List<Task> taskList)` Sets the task list. |
    | `protected void` | ClusteringEngine.`setTaskReceivedList(java.util.List<Task> taskReceivedList)` Sets the task received list. |
    | `protected void` | ClusteringEngine.`setTaskSubmittedList(java.util.List<Task> taskSubmittedList)` Sets the tasks submitted list. |
    | `void` | ClusteringEngine.`submitTaskList(java.util.List<Task> list)` This method is used to send to the broker the list of cloudlets. |
  - ### Uses of Task in org.workflowsim.clustering

    Methods in org.workflowsim.clustering that return Task

    | Modifier and Type | Method and Description |
    | --- | --- |
    |  |  |
    | --- | --- |
    | `Task` | BasicClustering.`addRoot()` |

    Methods in org.workflowsim.clustering that return types with arguments of type Task

    | Modifier and Type | Method and Description |
    | --- | --- |
    |  |  |
    | --- | --- |
    | `java.util.List<Task>` | BasicClustering.`getTaskList()` Gets the task list |
    | `java.util.List<Task>` | ClusteringInterface.`getTaskList()` get task list. |
    | `java.util.ArrayList<Task>` | TaskSet.`getTaskList()` Gets task list |

    Methods in org.workflowsim.clustering with parameters of type Task

    | Modifier and Type | Method and Description |
    | --- | --- |
    |  |  |
    | --- | --- |
    | `void` | TaskSet.`addTask(Task task)` Adds a task to this taskSet |
    | `protected Job` | BasicClustering.`addTasks2Job(Task task)` Add a task to a new job |

    Method parameters in org.workflowsim.clustering with type arguments of type Task

    | Modifier and Type | Method and Description |
    | --- | --- |
    |  |  |
    | --- | --- |
    | `void` | TaskSet.`addTask(java.util.ArrayList<Task> list)` Adds a list of task to this taskSet |
    | `void` | BasicClustering.`setTaskList(java.util.List<Task> list)` Sets the task list |
    | `void` | ClusteringInterface.`setTaskList(java.util.List<Task> list)` set the task list. |

- Overview
- Package
- Class
- Use
- Tree
- Deprecated
- Index
- Help

- Prev
- Next

- Frames
- No Frames

- All Classes
